# Activating PIK3CA mutation promotes overgrowth of adipose tissue via inhibiting lipophagy in macrodactyly

**DOI:** 10.1038/s41419-025-08024-x

**Published:** 2025-10-06

**Authors:** Yating Yin, Xiao Zhang, Shihui Lin, Zhibo Wang, Baoxing Tian, Xinyi Dai, Aiping Yu, Huixiao Li, Hailei Mao, Bin Wang

**Affiliations:** 1 Department of Plastic and Reconstructive Surgery, Shanghai Ninth People’s Hospital, Shanghai Jiao Tong University School of Medicine, Shanghai, China; 2 Shanghai Key Laboratory of Tissue Engineering, Shanghai Ninth People’s Hospital, Shanghai Jiao Tong University School of Medicine, Shanghai, China; 3 Orthopedics Department Ward 2, Northwest University First Hospital, Xi’an, China; 4 Department of Anesthesiology and Critical Care Medicine, Zhongshan Hospital, Fudan University, Shanghai, China

**Keywords:** Transcriptomics, Mesenchymal stem cells

## Abstract

Excessive proliferation and lipid accumulation of adipose tissue are the main pathological alterations in macrodactyly. Our previous studies found that macrodactyly exhibits abnormal lipid metabolism and inhibited autophagy, but the underlying mechanisms remain unclear. This study aims to investigate the regulatory mechanisms of autophagy in macrodactyly. The therapeutic impact and underlying mechanisms of autophagy on lipid accumulation, induced by a gain-of-function mutation of PIK3CA in macrodactyly, were assessed with respect to autophagy, lipid metabolism, oxidative stress, and deubiquitination. Autophagy deficiency resulting from PIK3CA mutation in macrodactyly led to excessive accumulation of adipose tissue. Lipid accumulation can be mitigated by inducing lipophagy of lipid droplets (LDs) in adipose derived stem cells of macrodactyly (Mac-ADSCs). The subsequent increase in free fatty acids (FFA) led to mitochondrial oxidative stress in Mac-ADSCs. Inducing autophagy exacerbated mitochondrial oxidative stress in Mac-ADSCs, thereby contributing to apoptosis. Additionally, the ablation of the deubiquitinase USP15 facilitated the degradation of LDs in Mac-ADSCs, through ubiquitin-dependent macrolipophagy. USP15 inhibitor reduced lipid accumulation in macrodactyly adipose tissue xenografts. In conclusion, activating PIK3CA mutation promotes excessive proliferation and lipid accumulation of Mac-ADSCs by inhibiting lipophagy. Targeted inhibition of USP15 may serve as a promising therapeutic approach for treating macrodactyly.

**Figure:**
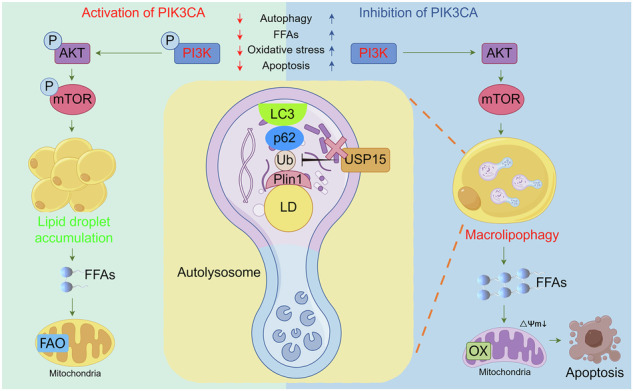
A schematic illustrates that activating PIK3CA mutation promotes overgrowth of adipose tissue via inhibiting lipophagy in macrodactyly.

A schematic illustrates that activating PIK3CA mutation promotes overgrowth of adipose tissue via inhibiting lipophagy in macrodactyly.

## Introduction

Macrodactyly is a rare nonhereditary congenital disorder characterized by the enlargement of one or multiple digits in the hand or foot compared to the contralateral or neighboring rays, often regarded as a benign tumor-like condition [1]. It can occur in isolation or as part of a syndrome, affecting various tissues, including bones, muscles, adipose tissue, and nerves, which may impair limb function and cause significant psychological stress for affected children and their families [2]. The prevalence of macrodactyly is difficult to determine, with estimates ranging from 1 in 200,000 (US) to 1 in 50,000 (Sweden) and some reports indicate a higher frequency in the lower limbs, from 1 in 50,000 to 1 in 18,000 [3]. Ongoing research is exploring the etiology of macrodactyly, particularly the role of somatic gain-of-function mutations in the PIK3CA gene, which are associated with the PIK3CA-Related Overgrowth Spectrum (PROS) and are not inherited [4, 5]. Tissue genetic mosaicism contributes to ongoing growth, and surgical treatment expectations have evolved to acknowledge that it cannot change the underlying growth patterns. The severity of mutations and their tissue specificity varies highly as they can occur at different times during embryogenesis, with macrodactyly developing later [6, 7]. Aggressive adipose tissue overgrowth is a hallmark of macrodactyly. Our previous study demonstrated that activating PIK3CA mutation promotes adipogenesis of adipose-derived stem cells (ADSCs) in macrodactyly [8]. Small molecule inhibitors targeting the PI3K/AKT/mTOR pathway reduced dermal fibroblast proliferation in PROS patients, suggesting potential for targeted therapy in this condition [9]. PI3Kα isoform-selective inhibitor Alpelisib (BYL719) impaired adipogenesis in ADSCs of macrodactyly [8]. However, the mechanism by which targeting PI3K inhibits the survival of ADSCs and lipid accumulation in macrodactyly remains unclear. Although targeting PI3K mutations is beneficial, it is insufficient to resolve fat accumulation in clinical patients. Therefore, we hypothesize that other regulatory pathways may also be involved.

Lipid accumulation is integral to the initiation and progression of macrodactyly. Lipid droplets (LDs) are essential organelles for lipid storage and metabolism, with their homeostasis maintained by the balance between lipid synthesis and hydrolysis [10, 11]. Autophagy is a lysosome-dependent cellular degradation pathway [12]. LDs are specifically recognized by the macroautophagic machinery and are effectively sequestered into autophagosomes, facilitating the release of free fatty acids (FFAs) in a process known as lipophagy [13]. Several essential components of the autophagy pathway are subject to ubiquitination [14, 15]. SQSTM1/p62 serves as a critical signaling hub and selective autophagy receptor, primarily mediated through its Phox1 and Bem1p (PB1) domain, ubiquitin-association (UBA) domain, and LC3-interacting region (LIR) [16]. Various deubiquitinating enzymes (DUBs) have been identified as modulators of autophagy by targeting components within the autophagy pathway for deubiquitination. Notably, ubiquitin specific peptidase 8 (USP8) has been shown to directly interact with and deubiquitinate SQSTM1, specifically removing ubiquitin moieties at lysine 420 (K420) [17]. Impaired autophagy has been documented to be associated with ectopic lipid accumulation in hepatocytes, which predisposes individuals to nonalcoholic fatty liver disease (NAFLD) [18]. Recent studies have demonstrated that USP15 plays a role in regulating hepatitis C viral RNA translation and lipid droplet formation in hepatocytes [19]. Our findings indicated that a deficiency in ubiquitin-dependent autophagy may contribute to excessive fat infiltration in macrodactyly.

## Results

### PIK3CA mutation upregulated lipid accumulation and downregulated autophagy in adipose tissue and adipose derived stem cells of macrodactyly

**Fig. 1:**
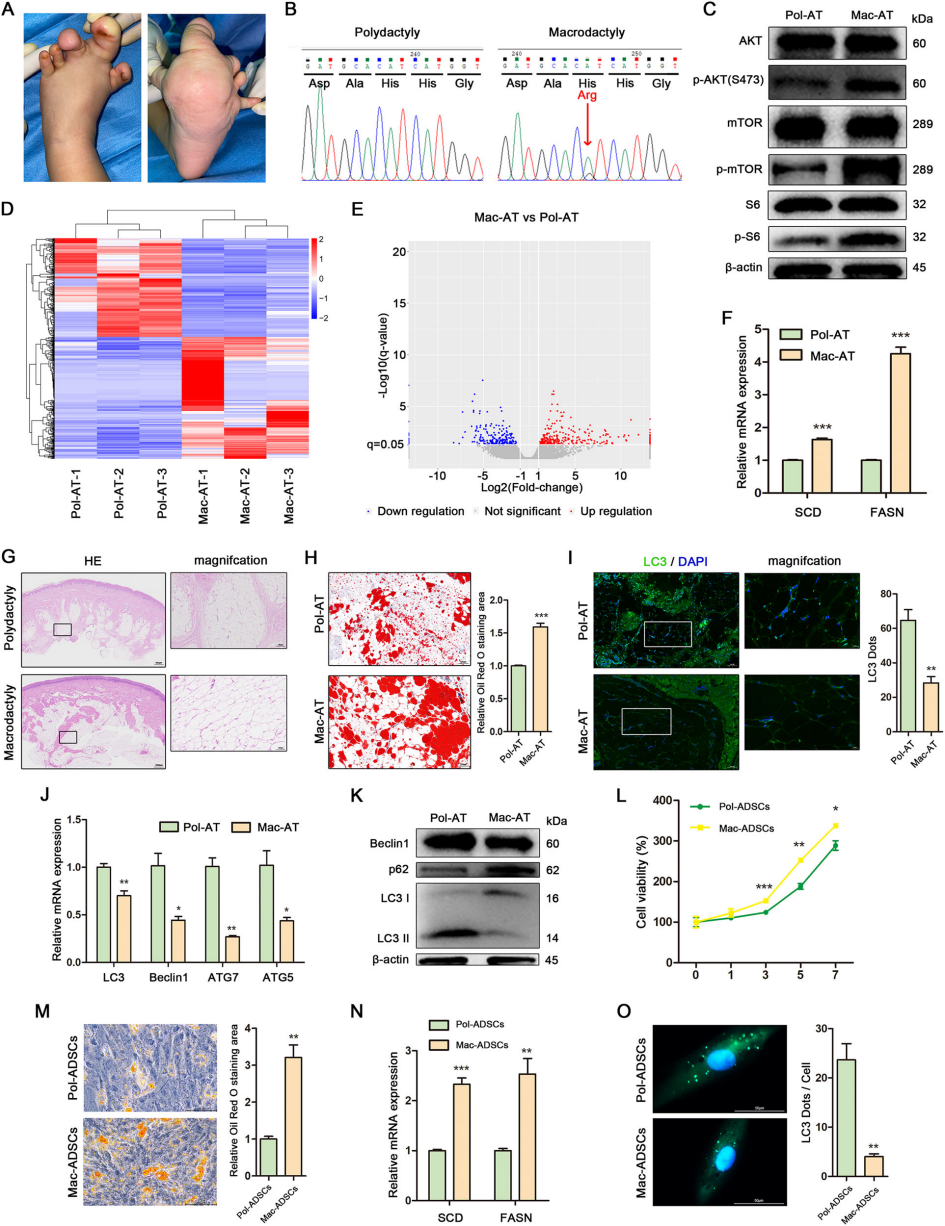
PIK3CA mutation upregulated lipid accumulation and downregulated autophagy in adipose tissue and adipose derived stem cells of macrodactyly. **A** Excessive accumulation of adipose tissue was observed in a patient with macrodactyly. **B** Sanger sequencing identified mutations in adipose tissue of polydactyly (Pol-AT) and macrodactyly (Mac-AT). **C** Western blot analysis of proteins in the PI3K/AKT/mTOR pathway in Pol-AT and Mac-AT. β-actin was used as an internal control. **D** Heat map representing color-coded expression levels of differentially expressed genes (DEGs) in Pol-AT and Mac-AT (GSE298035). **E** Volcano plot illustrating gene expression differences in Pol-AT and Mac-AT, with permutation p-values indicating significance. **F** RT-qPCR analysis of lipid metabolism-related mRNAs in Pol-AT and Mac-AT. **G** HE staining of Pol-AT and Mac-AT. Scale bar in the original images: 500 μm, 1000 μm. Scale bar in the magnified images: 100 μm. **H** Oil Red O staining and quantitative assessment of Pol-AT and Mac-AT. Scale bar: 100 μm. **I** Immunofluorescence staining targeting LC3 in Pol-AT and Mac-AT. Scale bar in the original images: 100 μm. Scale bar in the magnified images: 20 μm. Autophagic flux assessed by counting LC3 dots. **J** RT-qPCR analysis of autophagy-related mRNAs in Pol-AT and Mac-AT. **K** Western blot analysis of autophagy-related proteins in Pol-AT and Mac-AT. β-actin was used as an internal control. **L** Growth curves of adipose derived stem cells of polydactyly (Pol-ADSCs) and macrodactyly (Mac-ADSCs). **M** Oil Red O staining and quantitative assessment of Pol-ADSCs and Mac-ADSCs. Scale bar: 100 μm. **N** RT-qPCR analysis of lipid metabolism -related mRNAs in Pol-ADSCs and Mac-ADSCs. **O** Immunofluorescence staining targeting LC3 in Pol-ADSCs and Mac-ADSCs. Scale bar: 50 μm. Autophagic flux measured by counting LC3 dots per cell. Data are represented as mean ± SD of three independent experiments (n = 3). *p < 0.05, **p < 0.01, ***p < 0.001.

To investigate the pathological mechanisms underlying macrodactyly, we first examined the histological and molecular features of adipose tissue in affected individuals. An increase in soft tissue volume, particularly due to the accumulation of adipose tissue, was observed in macrodactyly (Fig. 1A). Given the known role of PIK3CA mutations in overgrowth syndromes, we hypothesized that such mutations might contribute to the adipose hypertrophy seen in macrodactyly. Sanger sequencing was employed to identify mutations in the adipose tissue of individuals with polydactyly (Pol-AT) and macrodactyly (Mac-AT). Notably, the PIK3CA mutation (c.3140 A>G, p.H1047R) was detected in macrodactyly, whereas it was absent in all cases of polydactyly (Fig. 1B). The elevated expression levels of phosphorylated AKT, mTOR, and S6 in Mac-AT was consistent with PI3K signaling hyperactivation, indicating the PI3K/AKT/mTOR pathway as a key phenotypic driver (Fig. 1C). To further investigate the pathological alterations associated with macrodactyly, we conducted RNA sequencing (RNA-Seq) analysis on Pol-AT and Mac-AT samples (Fig. 1D). Differential expression analysis identified 288 genes as upregulated and 233 genes as downregulated in Mac-AT compared to Pol-AT (Fig. 1E). The expression of lipid metabolism markers identified through RNA-Seq, including SCD and FASN, was consistently higher in Mac-AT than in Pol-AT, as confirmed by RT-qPCR analysis (Fig. 1F). These findings were corroborated by histological analyses. Histological staining with hematoxylin and eosin (HE) was performed on Pol-AT and Mac-AT samples. The adipocytes in macrodactylous tissue were observed to be larger than those in polydactylous tissue, with noticeable structural changes in the macrodactylous adipose tissue, suggesting increased hypertrophy of the adipocytes (Fig. 1G). Furthermore, lipid accumulation was significantly more pronounced in Mac-AT compared to Pol-AT (Fig. 1H). Furthermore, immunofluorescence staining for LC3 revealed a decrease in autophagic flux within Mac-AT (Fig. 1I). To further explore the impact of the PIK3CA mutation on autophagy in Mac-AT, we analyzed the mRNA and protein levels associated with autophagy. Our findings revealed a significant inhibition of autophagic flux in Mac-AT compared to Pol-AT (Fig. 1J, K). To determine whether these changes were intrinsic to adipose-derived stem cells (ADSCs), we isolated ADSCs from both polydactyly (Pol-ADSCs) and macrodactyly (Mac-ADSCs) samples. Both Pol-ADSCs and Mac-ADSCs exhibited similar surface ADSCs markers, being positive for CD29, CD90, and CD105, but negative for CD34, CD45, and CD106 (Fig. S1). However, the cell growth curve indicated a significantly higher proliferation rate in Mac-ADSCs compared to Pol-ADSCs (Fig. 1L). Following adipogenic differentiation, Mac-ADSCs exhibited significantly greater positive Oil Red O staining compared to Pol-ADSCs (Fig. 1M). Similarly, the expression levels of lipid metabolism markers were elevated in Mac-ADSCs relative to Pol-ADSCs (Fig. 1N). Additionally, autophagic flux was markedly inhibited in Mac-ADSCs compared to Pol-ADSCs (Fig. 1O). These results demonstrate that the PIK3CA mutation drives both hyperproliferation and metabolic reprogramming in Mac-ADSCs, contributing to the pathological adipose expansion in macrodactyly.

### Targeted inhibition of PIK3CA reduced proliferation and restored lipophagy in Mac-ADSCs

**Fig. 2:**
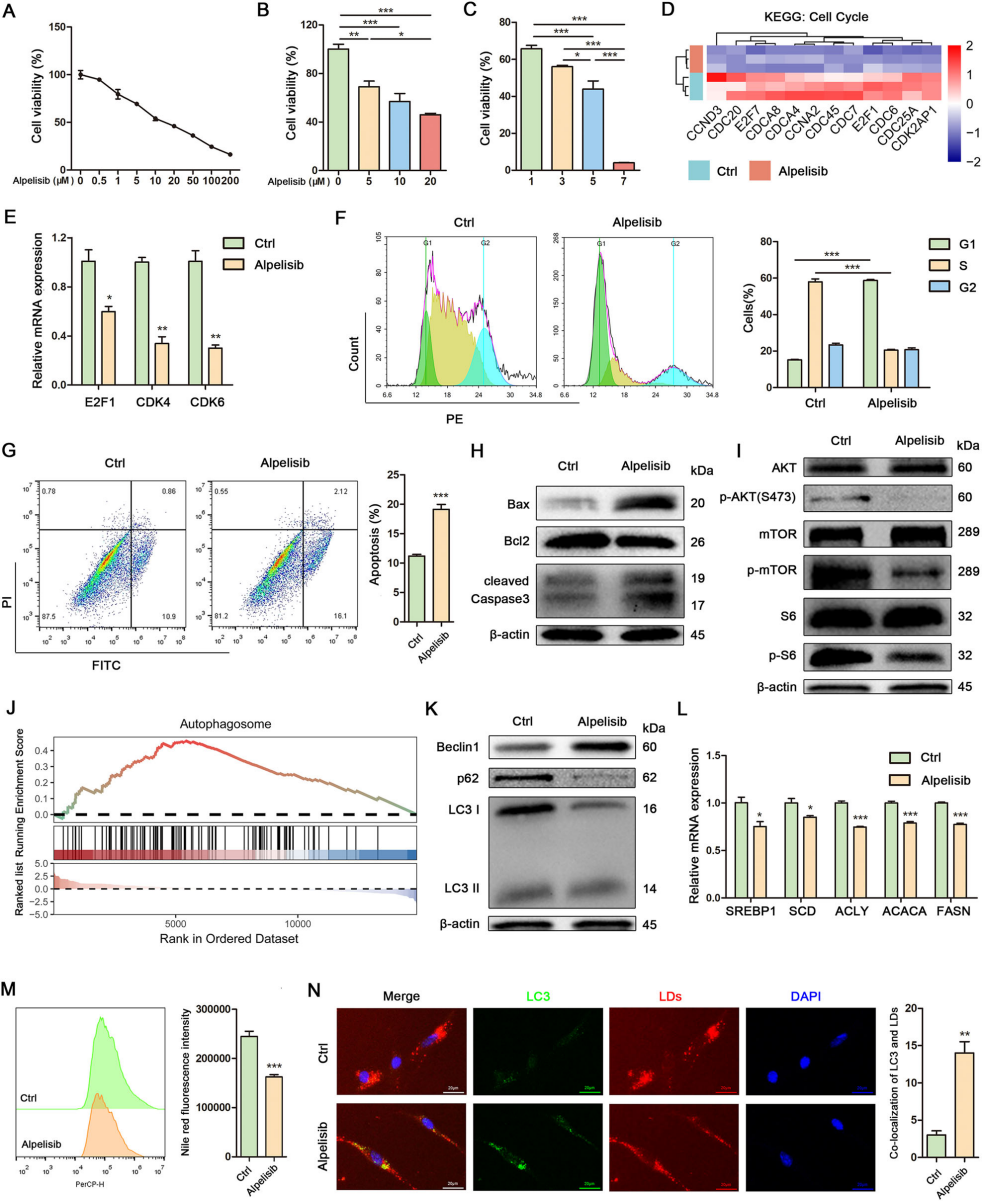
Targeted inhibition of PIK3CA reduced proliferation and restored lipophagy in Mac-ADSCs. **A** Cell viability was determined by CCK8 in Mac-ADSCs after 24-h treatment with Alpelisib (0.5 μΜ, 1 μΜ, 5 μΜ, 10 μΜ, 20 μΜ, 50 μM, 100 μM, 200 μM). **B** Cell viability was determined by CCK8 in Mac-ADSCs after 24-h treatment with Alpelisib (5 μΜ, 10 μM, 20 μM). **C** Cell viability was determined by CCK8 in Mac-ADSCs after 1, 3, 5, and 7 days of treatment with 10 μM Alpelisib. **D** A heatmap plot depicting the leading edge subset of genes involving in cell cycle pathway among DEGs between Pol-ADSCs and Mac-ADSCs (GSE151840). **E** RT-qPCR analysis of cell cycle-related mRNAs in Mac-ADSCs treated with or without Alpelisib. **F** Flow cytometry analysis and quantitative assessment of the cell cycle in Mac-ADSCs treated with or without Alpelisib. **G** Cell apoptosis was detected using the annexin V-FITC/PI kit. The early apoptotic cells (annexin V+/PI−) and late apoptotic cells (annexin V−/PI+) were analyzed in Mac-ADSCs treated with or without Alpelisib. **H** Western blot analysis of apoptosis-related proteins in Mac-ADSCs treated with or without Alpelisib. β-actin was used as an internal control. **I** Western blot analysis of PI3K/AKT/mTOR pathway proteins in Mac-ADSCs treated with or without Alpelisib. β-actin was used as an internal control. **J** GSEA plot showing enrichment of autophagosome pathway genes among DEGs between Pol-ADSCs and Mac-ADSCs (GSE151840). **K** Western blot analysis of autophagy-related proteins in Mac-ADSCs treated with or without Alpelisib. β-actin was used as an internal control. **L** RT-qPCR analysis of lipid metabolism-related mRNAs in Mac-ADSCs treated with or without Alpelisib. **M** Flow cytometry analysis and quantitative assessment of intracellular lipid levels in Mac-ADSCs treated with or without Alpelisib. **N** LDs (red) were visualized with Nile red staining. LC3 puncta (green) on LDs were visualized by immunofluorescence staining. Scale bar: 20 μm. Lipophagic flux measured by counting co-localization of LC3 and LDs in Mac-ADSCs. Data are represented as mean ± SD of three independent experiments (n = 3). *p < 0.05, **p < 0.01, ***p < 0.001.

Given the established role of PIK3CA hyperactivation in macrodactyly, the potential for pharmacological PIK3CA inhibition to reverse the observed phenotypes was subsequently investigated. Treatment with Alpelisib, a PIK3CA inhibitor, resulted in a dose-dependent decrease in cell viability at concentrations ranging from 0.5 μM to 200 μM (Fig. 2A, B). Furthermore, a time-dependent decrease in cell viability was observed with 10 μM Alpelisib (Fig. 2C). Mechanistically, KEGG analysis indicated that Alpelisib significantly inhibited the cell cycle pathway (Fig. 2D). Correspondingly, mRNAs associated with the cell cycle, including E2F1, CDK4, and CDK6, were markedly suppressed by Alpelisib (Fig. 2E). This finding was corroborated by flow cytometry analysis (Fig. 2F). Furthermore, cell apoptosis was assessed using flow cytometry and western blot, revealing that Alpelisib induced apoptosis in Mac-ADSCs (Fig. 2G, H). Additionally, Alpelisib markedly suppressed the activity of the PI3K/AKT/mTOR signaling pathway in Mac-ADSCs (Fig. 2I). These results confirmed the dependency of Mac-ADSCs on PI3K signaling for survival. Notably, the upregulation of the autophagosome pathway was observed in Mac-ADSCs treated with Alpelisib (Fig. 2J). Consistently, proteins related to autophagy were significantly upregulated by Alpelisib in Mac-ADSCs (Fig. 2K). The mRNA levels of lipid metabolism genes including SREBP1, SCD, ACLY, ACACA, and FASN, were downregulated in Mac-ADSCs treated with Alpelisib (Fig. 2L). These results indicate that PI3K inhibition activates lipophagy, as evidenced by Alpelisib-mediated reduction in lipid accumulation (Fig. 2M) and increased autophagosome formation co-localizing with lipid droplets (LDs) in Mac-ADSCs, suggesting enhanced LDs degradation (Fig. 2N). These findings demonstrate that PIK3CA inhibition not only curbs proliferation but also reinstates lipid turnover via autophagy.

### Lipophagy upregulation promotes mitochondrial oxidative stress via excessive FFAs accumulation in Mac-ADSCs

**Fig. 3:**
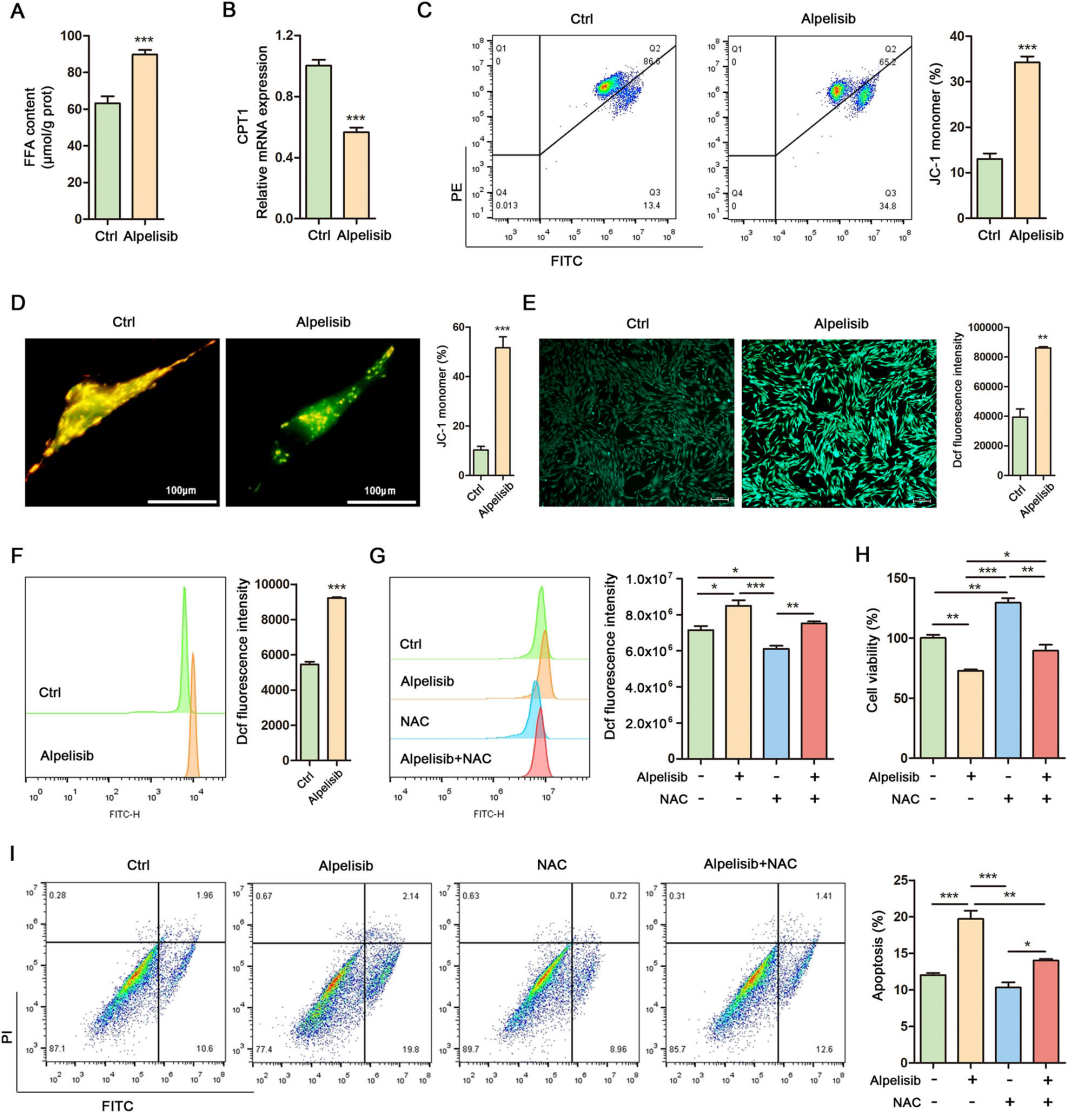
Lipophagy upregulation promotes mitochondrial oxidative stress via excessive FFAs accumulation in Mac-ADSCs. **A** Free fatty acids (FFAs) content was measured in Mac-ADSCs treated with or without Alpelisib. **B** RT-qPCR analysis of CPT1 mRNA in Mac-ADSCs treated with or without Alpelisib. **C** Flow cytometry analysis and quantitative assessment of JC-1 monomer generation in Mac-ADSCs treated with or without Alpelisib. **D** JC-1 monomers (green) and aggregates (orange) were imaged and quantitatively assessed in Mac-ADSCs treated with or without Alpelisib. **E** Intracellular ROS generation in Mac-ADSCs treated with or without Alpelisib was visualized and quantitatively analyzed. Scale bar: 50 μm. **F** Flow cytometry analysis and quantitative assessment of ROS generation in Mac-ADSCs treated with or without Alpelisib. **G** Flow cytometry analysis and quantitative assessment of ROS generation in Mac-ADSCs after Alpelisib (10 μM) or NAC (5 mM) treatment for 24 h. **H** Cell viability was determined by CCK8 in Mac-ADSCs after Alpelisib (10 μM) or NAC (5 mM) treatment for 24 h. **I** Flow cytometry analysis and quantitative assessment of cell apoptosis in Mac-ADSCs after Alpelisib (10 μM) or NAC (5 mM) treatment for 24 h. Data are represented as mean ± SD of three independent experiments (n = 3). *p < 0.05, **p < 0.01, ***p < 0.001.

The enhancement of lipophagy by Alpelisib facilitated the production of FFAs in Mac-ADSCs following a 10-day adipogenic induction period (Fig. 3A). However, the expression of carnitine palmitoyltransferase 1 (CPT1), a key enzyme regulating fatty acid oxidation, was observed to decrease rather than increase in response to elevated FFAs levels (Fig. 3B). We hypothesize that while lipophagy mediates lipid degradation, the consequent release of excessive FFAs could impair mitochondrial β-oxidation. Consequently, we assessed alterations in mitochondrial membrane potential using JC-1 staining. The results indicated that the induction of lipophagy led to depolarization of the mitochondrial membrane potential, as evidenced by an increased percentage of JC-1 monomers (Fig. 3C, D). To further explore whether the induction of lipophagy enhances mitochondrial oxidative stress, intracellular reactive oxygen species (ROS) production was assessed in Mac-ADSCs following 24 h of Alpelisib treatment, utilizing fluorescence probe detection and flow cytometry. The data revealed that Alpelisib treatment significantly increased intracellular ROS generation in Mac-ADSCs (Fig. 3E, F). Additionally, ROS generation induced by Alpelisib was effectively mitigated by the ROS inhibitor N-acetylcysteine (NAC) (Fig. 3G). The reduction in cell viability caused by Alpelisib in Mac-ADSCs was reversed upon treatment with NAC (Fig. 3H). Furthermore, NAC reversed the apoptosis induced by Alpelisib in Mac-ADSCs (Fig. 3I). These findings demonstrate that lipophagy activation leads to FFA-mediated mitochondrial oxidative stress in Mac-ADSCs.

### Inducing autophagy exacerbated mitochondrial oxidative stress and apoptosis in Mac-ADSCs

**Fig. 4:**
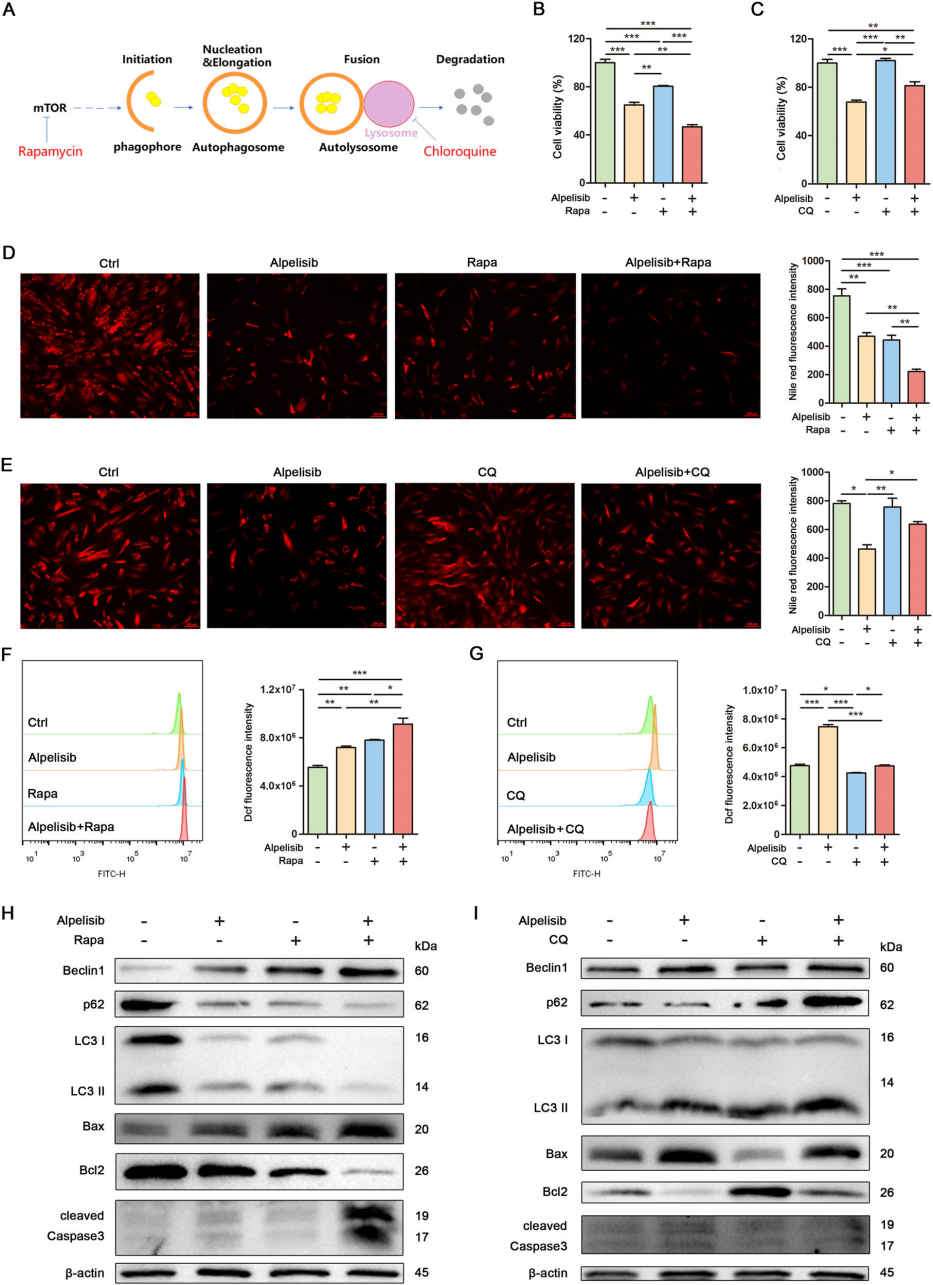
Inducing autophagy exacerbated mitochondrial oxidative stress and apoptosis in Mac-ADSCs. **A** A schematic illustrates the processes of autophagy initiation, elongation, autophagosome maturation, lysosomal fusion, and cargo degradation. **B** Cell viability was determined by CCK8 in Mac-ADSCs after Alpelisib (10 μM) or Rapamycin (5 μM) treatment for 24 h. Rapa: Rapamycin. **C** Cell viability was determined by CCK8 in Mac-ADSCs after Alpelisib (10 μM) or Chloroquine (5 μM) treatment for 24 h. CQ Chloroquine. **D** Intracellular lipid levels in Mac-ADSCs after Alpelisib (10 μM) or Rapamycin (5 μM) treatment for 24 h were visualized with Nile red staining and quantitatively analyzed. **E** Intracellular lipid levels in Mac-ADSCs after Alpelisib (10 μM) or Chloroquine (5 μM) treatment for 24 h were visualized with Nile red staining and quantitatively analyzed. **F** Flow cytometry analysis and quantitative assessment of ROS generation in Mac-ADSCs after Alpelisib (10 μM) or Rapamycin (5 μM) treatment for 24 h. **G** Flow cytometry analysis and quantitative assessment of ROS generation in Mac-ADSCs after Alpelisib (10 μM) or Chloroquine (5 μM) treatment for 24 h. **H** Western blot analysis of proteins related to autophagy or apoptosis in Mac-ADSCs after Alpelisib (10 μM) or Rapamycin (5 μM) treatment for 24 h. β-actin was used as an internal control. **I** Western blot analysis of proteins related to autophagy or apoptosis in Mac-ADSCs after Alpelisib (10 μM) or Chloroquine (5 μM) treatment for 24 h. β-actin was used as an internal control. Data are represented as mean ± SD of three independent experiments (n = 3). *p < 0.05, **p < 0.01, ***p < 0.001.

The schematic illustrates a model representing the current understanding of the processes involved in autophagy, including initiation, elongation, autophagosome maturation, fusion with the lysosome, and subsequent cargo degradation. (Fig. 4A). To further explore the role of autophagy in oxidative stress and apoptosis, we employed Rapamycin (an inducer) and Chloroquine (an inhibitor) to modulate autophagic activity in Mac-ADSCs. Our data indicated that Rapamycin enhanced Alpelisib-induced cell death, whereas Chloroquine mitigated this effect (Fig. 4B, C). The reduction in intracellular lipid content caused by Alpelisib was enhanced by Rapamycin and diminished by Chloroquine (Fig. 4D, E). Moreover, the intracellular generation of ROS induced by Alpelisib was intensified by Rapamycin and attenuated by Chloroquine (Fig. 4F, G). Furthermore, apoptosis triggered by Alpelisib was intensified by Rapamycin and mitigated by Chloroquine (Fig. 4H, I). These findings demonstrate that Mac-ADSCs display deficient lipophagic flux, in which pharmacologically induced excessive autophagy triggers mitochondrial oxidative stress and apoptotic cell death.

### The targeted inhibition of USP15 facilitated ubiquitin-dependent macrolipophagy and decreased lipid accumulation in Mac-ADSCs

**Fig. 5:**
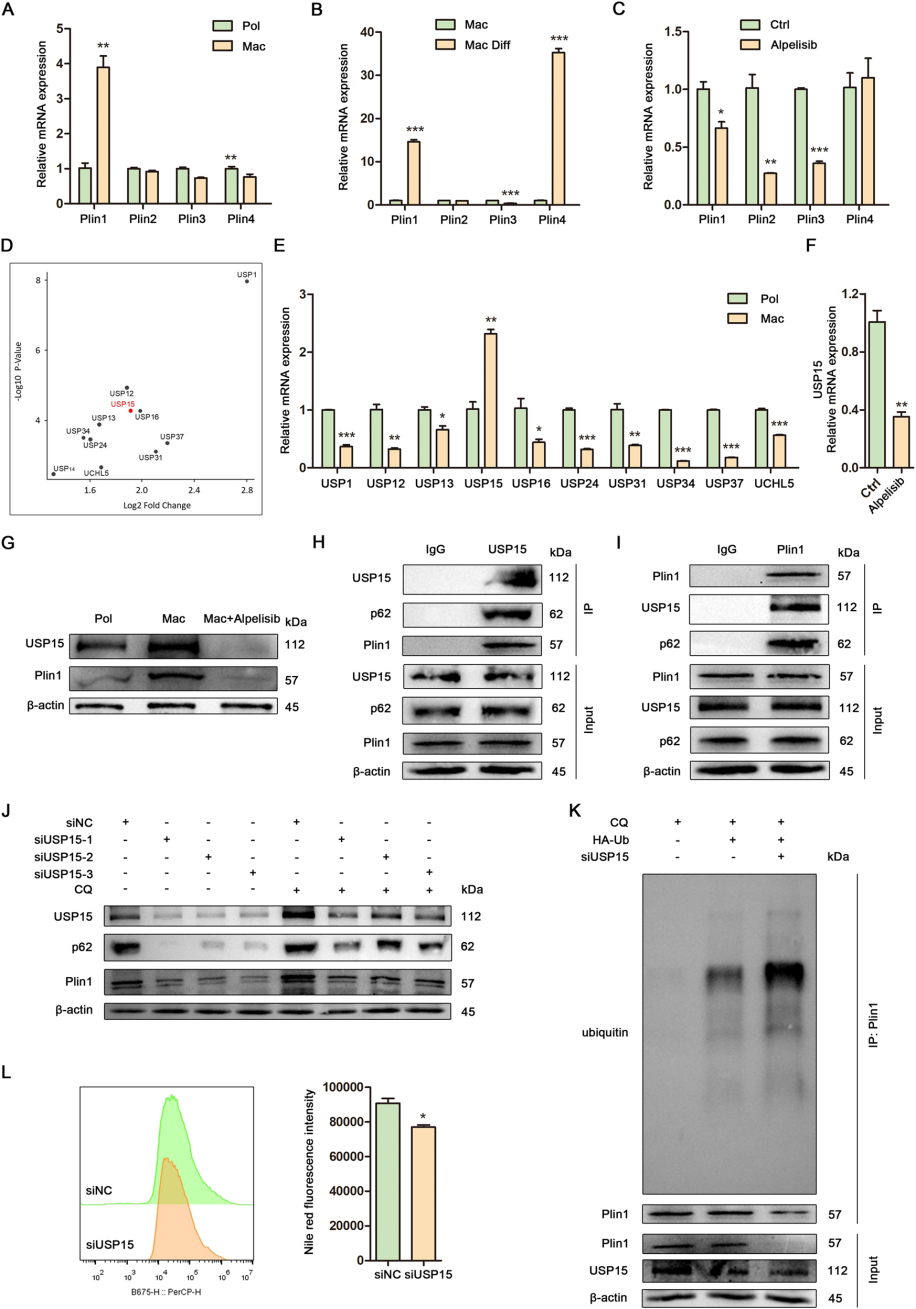
The targeted inhibition of USP15 facilitated ubiquitin-dependent macrolipophagy and decreased lipid accumulation in Mac-ADSCs. **A** RT-qPCR analysis of Perilipin mRNAs in Pol-ADSCs and Mac-ADSCs. **B** RT-qPCR analysis of Perilipin mRNAs in Mac-ADSCs after adipogenic induction for 24 h. **C** RT-qPCR analysis of Perilipin mRNAs in Mac-ADSCs treated with or without Alpelisib. **D** Volcano plot depicting the differences in the expression of upregulated genes involved in deubiquitination among Pol-ADSCs and Mac-ADSCs (GSE151840). **E** RT-qPCR analysis of deubiquitination-related mRNAs in Pol-ADSCs and Mac-ADSCs. **F** RT-qPCR analysis of USP15 mRNA in Mac-ADSCs treated with or without Alpelisib. **G** Western blot analysis of USP15 and Plin1 expression in Pol-ADSCs, Mac-ADSCs and Mac-ADSCs treated with Alpelisib. β-actin was used as an internal control. **H** Co-IP assay using IgG and USP15 antibodies detects the interactions between p62, Plin1 and USP15 in Mac-ADSCs. β-actin was used as an internal control. **I** Co-IP assay using IgG and Plin1 antibodies detects the interaction between USP15, p62 and Plin1 in Mac-ADSCs. β-actin was used as an internal control. **J** Mac-ADSCs were transfected with siRNAs against USP15 or control vector, and treated with or without Chloroquine. Western blot analysis detects effects of USP15 knockdown on the protein stability of p62 and Plin1. β-actin was used as an internal control. **K** Western blot analysis of deubiquitination assays of Plin1. Mac-ADSCs were transfected with siRNA against USP15 and/or HA-Ub plasmid followed by Chloroquine treatment. β-actin was used as an internal control. **L** Flow cytometry analysis and quantitative assessment of intracellular lipid levels in Mac-ADSCs treated with siRNA against USP15 or control vector. Data are represented as mean ± SD of three independent experiments (n = 3). *p < 0.05, **p < 0.01, ***p < 0.001.

LDs are enveloped by one or more of the five perilipin family proteins. Given the central role of LDs proteins in lipophagy, we investigated the expression levels of the five perilipin genes (Plin1, Plin2, Plin3, Plin4, Plin5) in Mac-ADSCs. Our findings revealed that Plin1 was significantly upregulated in Mac-ADSCs compared to Pol-ADSCs, with its expression further increasing during adipogenic differentiation. This indicates that Plin1 may play a pivotal role in the formation and stabilization of LDs in Mac-ADSCs. It is noteworthy that RT-qPCR did not detect Plin5 expression in either Pol-ADSCs or Mac-ADSCs, suggesting that Plin5 may not be expressed in these cell types (Fig. 5A, B). Furthermore, the expression of Plin1 was reduced following Alpelisib treatment in Mac-ADSCs (Fig. 5C). Given that autophagic flux was enhanced post-Alpelisib treatment in Mac-ADSCs and considering that Plin1 serves as a substrate for selective autophagy, we propose the hypothesis that Plin1 undergoes degradation via selective autophagy to promote lipid hydrolysis in response to Alpelisib treatment. Since ubiquitination regulates selective autophagy and ubiquitin mediated proteolysis was identified among the top 10 enriched KEGG pathways in Mac-AT versus Pol-AT transcriptomes (Fig. S2), we screened for deubiquitinating enzymes. The volcano plot illustrates the differential expression of genes associated with deubiquitination between Pol-ADSCs and Mac-ADSCs (Fig. 5D). Among the deubiquitination genes analyzed, USP15 exhibited significantly higher expression levels in Mac-ADSCs compared to Pol-ADSCs (Fig. 5E), and its expression was notably reduced following Alpelisib treatment (Fig. 5F). Consistent findings were observed at the protein level through western blot analysis. Both Plin1 and USP15 were expressed at elevated levels in Mac-ADSCs relative to Pol-ADSCs, with their expression being downregulated by Alpelisib treatment (Fig. 5G). Based on these results, we proposed a hypothesis that USP15 may play a critical role in maintaining Plin1 protein stability by modulating selective autophagy. Co-IP assays revealed that USP15 directly interacts with the autophagy receptor p62 and the lipid metabolism-related protein Plin1 (Fig. 5H, I). To investigate the effects of USP15 knockdown on the protein stability of p62 and Plin1, Mac-ADSCs were transfected with siRNA targeting USP15 and subsequently treated with or without chloroquine, a lysosomal inhibitor. The results indicated that the protein degradation of p62 and Plin1 was enhanced following the downregulation of USP15 via siRNA (Fig. 5J). To further substantiate the role of USP15 in mediating the deubiquitination of Plin1, Mac-ADSCs were subjected to transfection with ubiquitin plasmid and/or siRNA targeting USP15, followed by treatment with Chloroquine. These findings indicated an increase in the polyubiquitination of Plin1 upon the suppression of USP15 in Mac-ADSCs (Fig. 5K). Since autophagy deficiency led to lipid accumulation in Mac-ADSCs, we investigated whether restoring lipophagy via USP15 inhibition could reverse this phenotype. It was observed that the knockdown of USP15 resulted in a reduction of lipid content in Mac-ADSCs (Fig. 5L). These findings indicate that USP15 potentially functions as a target in ubiquitin-mediated selective autophagy, thereby regulating lipid homeostasis in Mac-ADSCs.

### The pharmacological inhibition of USP15 reduced lipid accumulation in macrodactyly adipose tissue xenografts

**Fig. 6:**
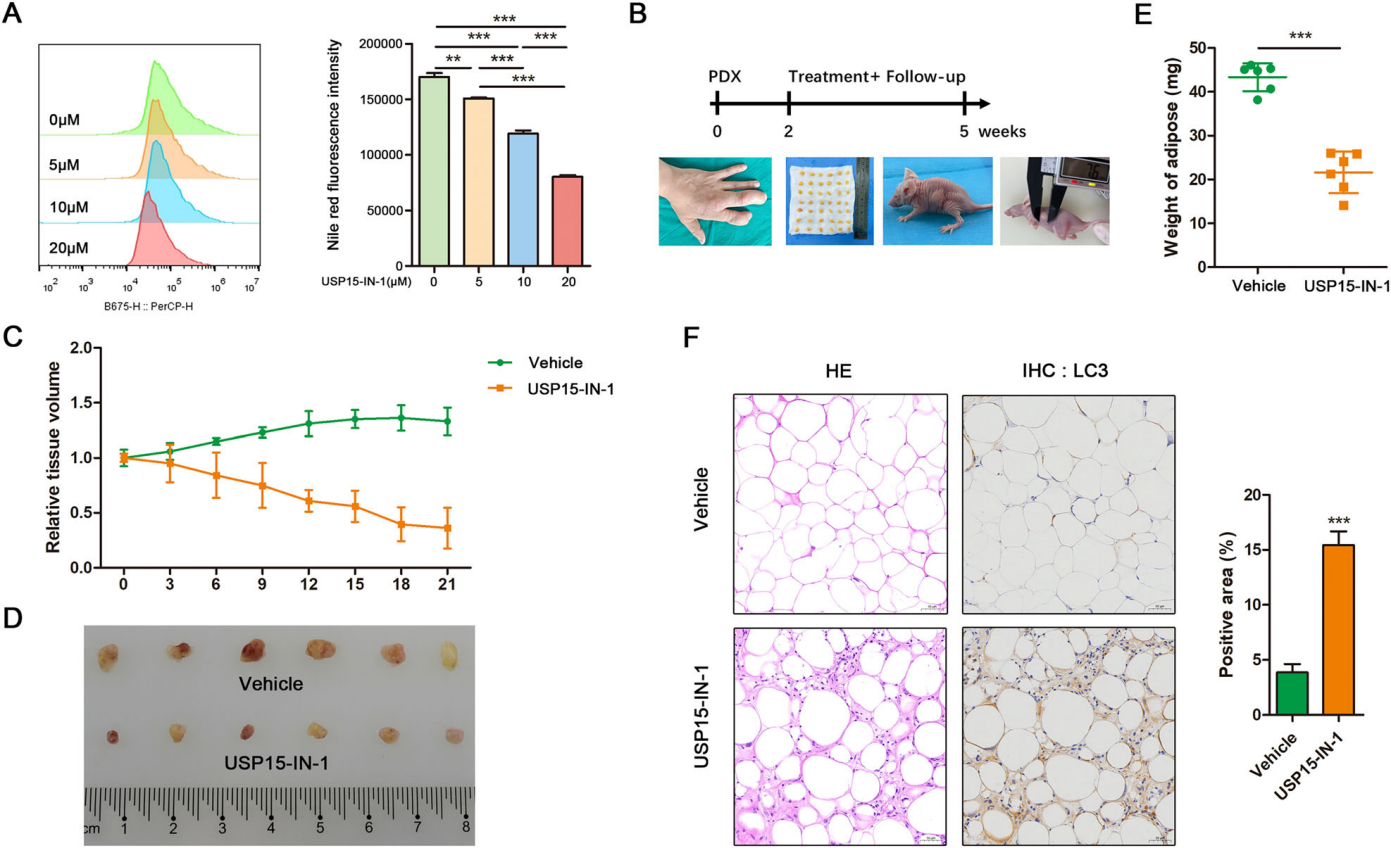
The pharmacological inhibition of USP15 reduced lipid accumulation in macrodactyly adipose tissue xenografts. **A** Flow cytometry analysis and quantitative assessment of intracellular lipid levels in Mac-ADSCs treated with USP15-IN-1, a USP15 inhibitor (5 μM, 10 μM, 20 μM). Data are represented as mean ± SD of three independent experiments (n = 3). **p < 0.01, ***p < 0.001. **B** PDX model experimental scheme. **C** Relative tissue volume of macrodactyly xenografts after 21 days’ USP15-IN-1 or vehicle treatment. **D** Image of macrodactyly xenografts from PDX models after 21 days’ USP15-IN-1 or vehicle treatment. **E** Weight of macrodactyly xenografts after 21 days’ USP15-IN-1 or vehicle treatment. **F** HE staining of macrodactyly xenografts treated with USP15-IN-1 or vehicle on day 21 (left). Immunohistochemical staining targeting LC3 of macrodactyly xenografts treated with USP15-IN-1 or vehicle on day 21 (right). Scale bar: 50 μm. Data are represented as mean ± SD of six independent biological replicates (n = 6). ***p < 0.001.

Treatment of Mac-ADSCs with USP15-IN-1, a USP15-specific inhibitor (5 μM, 10 μM, 20 μM) significantly reduced intracellular lipid accumulation in a dose-dependent manner (Fig. 6A). To evaluate the therapeutic potential of USP15 inhibition in vivo, we established patient-derived xenograft (PDX) models using macrodactyly adipose tissue (Fig. 6B). 21 days of USP15-IN-1 treatment significantly reduced both the volume (Fig. 6C, D) and weight (Fig. 6E) of xenografts compared to vehicle-treated controls. Immunohistochemical analysis demonstrated enhanced autophagic flux in USP15-IN-1-treated xenografts, as indicated by elevated LC3B expression (Fig. 6F). These findings establish that pharmacological USP15 inhibition attenuates pathological lipid accumulation in macrodactyly through activation of lipophagy.

## Discussion

Macrodactyly, a component of the PIK3CA Related Overgrowth Spectrum, presents a significant clinical challenge for physicians. Concurrently, it provides a valuable opportunity to investigate the morphology, histology, and, more recently, the molecular mechanisms underlying these conditions [20]. Our study established a previously unrecognized connection between PIK3CA mutation, ubiquitin-mediated autophagy regulation, and lipid metabolic reprogramming in Macrodactyly.

Macroautophagy represents a highly conserved lysosomal degradation pathway distinct from microautophagy and chaperone-mediated autophagy (CMA). This process uniquely requires the formation of double-membrane vesicles, known as autophagosomes, which encapsulate intracellular constituents such as proteins, macromolecular complexes, organelles, and even invading pathogens [21]. The initiation of autophagosome formation during autophagy is mediated by the activation of the ULK1/Atg1 kinase complex [22], which integrates upstream molecular signals primarily through two pivotal kinases: mTORC1 and PRKA/AMPK [23, 24]. The sterol regulatory element-binding protein 1 (SREBP1), which modulates the expression of several genes involved in lipid synthesis, such as ATP citrate lyase (ACLY), acetyl-CoA carboxylase alpha (ACACA), fatty acid synthase (FASN), and stearoyl-CoA desaturase (SCD), has been identified as a downstream target of mechanistic target of mTORC1 signaling [25, 26]. Regarding the degradation of LDs, in addition to the hydrolysis of triglycerides (TG) by lipases [27], LDs can also be sequestered by autophagosomes for subsequent lysosomal degradation, a process known as lipophagy [28]. Our RNA-seq and functional analyses revealed that PIK3CA-mutant Mac-ADSCs develop a unique metabolic dependency on both enhanced lipid synthesis and suppressed lipophagy. This dual perturbation creates a self-reinforcing cycle of lipid accumulation in Macrodactyly.

FFAs are transported from LDs to mitochondria to provide energy for β-oxidation. Vascular oxidative stress induced by FFAs may play a role in endothelial dysfunction observed in patients with insulin resistance [29]. Melatonin has been shown to mitigate palmitic acid-induced mitochondrial dysfunction by decreasing oxidative stress and promoting autophagy in bovine endometrial epithelial cells [30]. LBL21 induces a rapid depletion of intracellular glutathione (GSH), resulting in abnormal accumulation of ROS and mitochondrial dysfunction, as evidenced by a reduction in mitochondrial respiration and transmembrane potential [31]. The overexpression of ATG14 induced endoplasmic reticulum (ER) stress due to the accumulation of FFAs mediated by lipophagy, which subsequently resulted in ROS-dependent mitochondrial stress, ultimately leading to apoptosis [32]. Our findings demonstrated that excessive lipophagy induced apoptotic cell death in Mac-ADSCs through FFAs-mediated mitochondrial oxidative stress.

A variety of proteins continuously interact with the surface of LDs, notably members of the perilipin (Plin) protein family: Plin1 to Plin5 [33]. Plin1 and Plin4 are mainly expressed in white adipose tissue, while Plin2 and Plin3 are ubiquitously expressed. In contrast, Plin5 is primarily expressed in cardiac and skeletal muscles, brown adipose tissue, and the liver [11]. During the early stages of steatosis, small LDs begin to accumulate in hepatocytes, coated by perilipins Plin2, Plin3, and Plin5. Subsequently, under conditions of chronic lipid overload, these perilipins are replaced by Plin1 as the lipid droplets increase in size, which is a hallmark of LDs maturation and macrovesicular steatosis [34]. Ubiquitination critically regulates multiple autophagy stages, significantly influencing cancer progression and treatment response [35]. Plin1 has been reported to be regulated by polyubiquitination, and ubiquitinated Plin1 may serve as a target for recognition by SQSTM1/p62, thereby promoting macrolipophagy [36, 37]. Conjugation of ubiquitin to substrate proteins leads to proteasomal degradation and can be reversed by deubiquitinating enzymes [38]. Notably, USP family deubiquitinases enhances the tolerance of tumor cells to chemotherapeutic agents by promoting autophagy [39]. USP9X contributes to autophagy impairment via Raptor stabilization in neurodegenerative mice [40]. Deubiquitinase USP1 enhances CCAAT/enhancer-binding protein beta (C/EBPβ) stability and accelerates adipogenesis and lipid accumulation [41]. Ubiquitinated substrates accumulate following USP36 inactivation may then be selectively recognized and aggregated by SQSTM1/p62, and ultimately targeted to autophagosomes [42]. Ubiquitin C-terminal hydrolase L1 (UCHL1), a deubiquitinating enzyme stabilizes Plin2 and thus lipid storage in skeletal muscle [43]. Acute free cholesterol accumulation induces ubiquitin- and autophagy-mediated degradation of Plin2 in hepatocytes [44]. Our results reveal that the USP15-Plin1-p62 interaction complex represents a potential mechanism for modulating LDs in Mac-ADSCs. Specifically, inhibitors targeting specific DUBs, such as USP1, USP7, USP14, and USP30 have shown promise in preclinical and clinical studies for cancer therapy [45]. Our findings demonstrate that USP15 inhibitor significantly reduces adipose graft volume and weight while enhancing autophagic flux in macrodactyly adipose PDX models. These results suggest that ubiquitination-dependent regulation of autophagy substrates may serve as a key mechanism governing lipophagy in Mac-ADSCs, potentially driving lipid metabolic reprogramming in macrodactyly.

The study does have limitations. Our investigation focused exclusively on the H1047R mutation, which although represents the most prevalent PIK3CA variant in macrodactyly, does not account for potential functional differences associated with other activating mutations. While PIK3CA mosaic activating mutations uniformly hyperactivate the PI3K signaling cascade in macrodactyly, they may exert mutation-specific effects on downstream processes, including potential variations in lipophagy impairment based on mutation loci and allele frequencies. Moreover, although the xenograft model provides valuable data, it cannot fully replicate human digit morphology. Additionally, the chronic effects of USP15 inhibition on adipose tissue remodeling and systemic metabolic homeostasis warrant further longitudinal studies.

In conclusion, this study demonstrated that the activation of PIK3CA mutation inhibited lipophagy, resulting in lipid accumulation in Mac-ADSCs. USP15-mediated Plin1 stabilization as a critical mechanism linking PIK3CA mutations to defective lipophagy in macrodactyly. By delineating this ubiquitin-dependent regulatory axis, we provide not only mechanistic insights into overgrowth pathogenesis but also a preclinical rationale for targeted therapeutic intervention.

## Materials and methods

### Patient information

Surgically amputated digits of macrodactyly patients and polydactyly patients were obtained from Department of Plastic and Reconstructive Surgery, Shanghai Ninth People’s Hospital. All protocols involving human subjects were reviewed and approved by the Institutional Review Board of Shanghai Ninth People’s Hospital (SH9H-2022-T357-1). All procedures were carried out in accordance with guidelines set forth by Declaration of Helsinki. Written informed consent was obtained from all participants.

### DNA isolation and sequencing

Genomic DNA was extracted from adipose tissue using the QIAamp DNA Mini Kit (QIAGEN, Germany, 51304). DNA concentrations were measured by NanoDrop spectrophotometer (Thermo Fisher, USA). Conventional PCR was performed using a Veriti thermocycler (Thermo Fisher, USA), and the amplified PCR products were analyzed by GENEWIZ (China) for Sanger sequencing.

### Western blot

Cell extracts were separated on SDS-polyacrylamide gel electrophoresis, then proteins were transferred to a nitrocellulose membrane and incubated with the following antibodies: rabbit monoclonal against AKT (1:1000; CST, USA, 4691), p-AKT (1:1000; CST, USA, 4058), mTOR (1:1000; CST, USA, 2983), p-mTOR(1:1000; CST, USA, 5536), S6 (1:1000; CST, USA, 2217), p-S6 (1:1000; CST, USA, 4858), Beclin1(1:1000; CST, USA, 3738), p62 (1:1000; CST, USA, 5114), LC3B(1:1000; CST, USA, 12741), cleaved Caspase3 (1:1000; CST, USA, 9664), β-actin(1:5000; Abclone, China, AC026); mouse monoclonal against Bax (1:1000; Santa Cruz, USA, sc-80658), Bcl2 (1:1000; Santa Cruz, USA, sc-56018), Plin1 (1:1000; Santa Cruz, USA, sc-390169), USP15 (1:1000; Santa Cruz, USA, sc-100629), and ubiquitin recombinant antibody (Proteintech, China, 80992). Immunoreactive protein bands were detected using Servicebio scanning system (China). The full length uncropped original western blots are shown in Figs. S3–5.

### RNA-sequencing analysis

Total RNA from Mac-AT and Pol-AT was purified using the RNeasy mini kit (QIAGEN, Germany, 74104). cDNA library preparation and sequencing were conducted following the Illumina standard protocol. Differential expression analysis utilized the edgeR package, identifying significantly modulated genes with an absolute log2 fold change >1 and a q-value < 0.05. The identified genes were further analyzed for GO, KEGG, and GSEA using the clusterProfiler package.

### RNA extraction and RT-qPCR

Total RNA extracted from cells or tissues with NcmSpin Cell/Tissue Total RNA Kit (NCM, China, M5105) was subjected to generate cDNA using PrimeScript RT Master Mix Kit (TaKaRa, Japan, RR036A). RT-qPCR was performed with TB Green Premix Ex Taq (TaKaRa, Japan, RR420A), using specific primers listed in Table S1. All specific primers were obtained from Servicebio (China). Relative gene expression was examined using the 2^−ΔΔCT^ method.

### HE staining

Tissue sections were deparaffinized, hydrated, and stained with hematoxylin for 5 min and eosin for 1 min, followed by dehydration and mounting.

### Oil Red O staining

Fresh frozen adipose tissue sections were fixed in 4% paraformaldehyde, stained with Oil Red O for 15 min, and counterstained with hematoxylin. Quantitative analysis of the Oil Red O stain was performed by ImageJ software (National Institutes of Health, USA).

### Immunofluorescence staining

Formalin-fixed paraffin-embedded tissue was deparaffinized, rehydrated, and subjected to heat-induced antigen retrieval, followed by block with 3% BSA. Cells on coverslips were fixed with paraformaldehyde, permeabilized with Triton-X 100, and blocked with BSA. After overnight incubation with rabbit monoclonal anti-LC3B antibody (1:100; Abcam, USA, ab192890), sections were incubated with Alexa Fluor 488-conjugated secondary antibody (1:1000; Abcam, USA, ab150077) and counterstained with DAPI (Yeasen, China, 40728ES). Images were acquired using a confocal microscope (Leica, Germany).

### Isolation and culture of human ADSCs

Tissue samples were minced, washed, and digested with an equal volume of 0.1% w/v collagenase NB4 (Nordmark, Germany, S1745401) in DMEM for 1 h at 37 °C. After centrifugation, the cell pellet was filtered and plated in hADSCs complete culture medium (Cyagen, China, HUXMD-90011). The medium was changed after 24–48 h, and cells were passaged upon reaching 80% confluence.

### Cell viability assay

Cell viability was assessed using the CCK8 assay (Beyotime, China, C0038) with absorbance at 450 nm reported as a percentage of control cells using a microplate reader (Molecular Devices, USA).

### Adipogenic differentiation

Cells were incubated in adipogenic culture medium according to the manufacturer’s instructions (Cyagen, China, HUXMD-90031).

### Drug treatments

ADSCs were treated with Alpelisib (MCE,China, HY-15244) (0.5 μM, 1 μM, 5 μM,10 μM, 20 μM, 50 μM, 100 μM, and 200 μM), NAC (MCE, China, HY-134495) (5 mM), Rapamycin (MCE, China, HY-10219) (5 μM), Chloroquine (MCE, China, HY-17589A) (5 μM) or USP15-IN-1 (MCE, China, HY-148046).

### Cell cycle flow cytometric analysis

Cells were fixed in ethanol, treated with RNase A and PI, then analyzed using a flow cytometer (Agilent, USA) according to the manufacturer’s protocol (KeyGEN, China, KGA9101).

### Apoptosis assay

Cells were stained with Annexin V-FITC/PI (Servicebio, China, G1511) and analyzed by flow cytometry to determine the apoptosis rate.

### Nile red staining

Cells were fixed and then incubated with Nile red (1 μg/ml) for 10 min at 37 °C according to the manufacturer’s protocol (Solarbio, China, N8440). Cellular Nile red-stained LDs were observed using fluorescence microscopy, and quantitated with flow cytometer with excitation and emission wavelength at 530 nm and 590 nm, respectively.

### Lipolysis assay

Lipolysis assay was performed by using the Free Fatty Acids (FFAs) assay Kit (Nanjing Jiancheng, China, A042-2-1) according to the manufacturer’s protocol.

### Mitochondrial transmembrane potential (ΔΨm)

Cells were incubated with JC-1 (Servicebio, China, G1515) to assess membrane potential. Flow cytometry (Agilent, USA) quantified monomers, while fluorescence microscopy (Zeiss, Germany) visualized green and orange fluorescence indicative of depolarization and polarization.

### Measurement of intracellular ROS

Cells were treated with 10 μM fluorescent probe CM-H2DCFDA (Solarbio, China, CA1410) and then detected using fluorescence microscope (Zeiss, Germany) and flow cytometry (Agilent, USA).

### Immunoprecipitation

Cell lysates were immunoprecipitated with IgG (Proteintech, China, 66360), USP15 (Santa Cruz, USA, sc-100629) or Plin1 (Santa Cruz, USA, sc-390169) antibody and protein A/G agarose beads (Santa Cruz, USA, sc-2003). The immunoprecipitates were washed, boiled in SDS sample buffer, and analyzed by Western blot after centrifugation.

### siRNAs and plasmids transfection

All siRNAs and plasmids were purchased from Hanbio (China). Cells were transfected with siRNA or plasmid using Lipofectamine 2000 (Invitrogen, USA, 11668019), following the manufacturers’ instructions. Gene-specific siRNAs sequences are listed in Table S2.

### PDX model establishment and drug treatment

All animal experiments were approved by Shanghai Ninth People’s Hospital Animal Experimentation Ethics Committee. Fresh macrodactyly adipose tissue from surgical specimens was minced into 2 × 2 × 3 mm^3^ fragments in cold DMEM (1% penicillin/streptomycin) and subcutaneously transplanted into 3–5-week-old female BALB/c nude mice (Shanghai JSJ Laboratory Animal Company, China). After a 2-week phase, mice were randomized into two groups (n = 6/group) receiving daily intraperitoneal injections for 21 days. Vehicle: 5% DMSO (TargetMol, China, T0341) + 40% PEG300 (TargetMol, China, T7022) + 5% Tween 80 (TargetMol, China, T13947) + 50% saline; USP15-IN-1 (MCE, China, HY-148046): 5 mg/kg in vehicle. Tumor volume was calculated as (length × width^2^)/2 using calipers. Mice were euthanized at week 5, with grafts harvested for photograph, weight measurement, histological and molecular analysis.

### Immunohistochemical staining

Paraffin sections were deparaffinized, rehydrated, and subjected to antigen retrieval in citrate buffer (pH 6.0). After blocking with 3% H₂O₂ and BSA, slides were incubated with rabbit monoclonal anti-LC3B antibody (1:100; Abcam, USA, ab192890) at 4 °C overnight, followed by HRP-conjugated secondary antibody (RT, 1 h). Signal was developed with DAB, counterstained with hematoxylin, and mounted for imaging.

### Statistical analysis

In vitro results are presented as mean ± standard deviations (SD) of three independent experiments. In vivo results are presented as mean ± SD of six independent biological replicates. Homogeneity of variance was tested before statistical analysis. Data were analyzed using unpaired *t*-test for two-group comparisons or one-way ANOVA for multiple comparisons, via SPSS software (SPSS 16.0, USA). *p*-value < 0.05 was considered statistically significant (*p < 0.05, **p < 0.01, and ***p < 0.001).

## Supplementary information

Supplementary materials

Figure S1

Figure S2

Figure S3

Figure S4

Figure S5

Table S1

Table S2

## Data Availability

The data that support the findings of this study are available from the corresponding author upon reasonable request.
