# Supplementary material for: Activating PIK3CA mutation promotes overgrowth of adipose tissue via inhibiting lipophagy in macrodactyly
Source: Cell Death Dis. 2025 Oct 6;16(1):686. doi: 10.1038/s41419-025-08024-x (PMC12501352; doi:10.1038/s41419-025-08024-x)
Supplement: Supplementary file 1 — Supplementary materials [file 41419_2025_8024_MOESM1_ESM.docx]

**Supplementary Materials and methods**

**Cell surface markers staining and flow cytometry**

Cell surface marker expression was analyzed using fluorochrome-conjugated anti-human antibodies: CD34 (Biolegend, USA, 343603), CD45 (Biolegend, USA, 304006), CD106 (eBioscience, USA, 53-1069-42), CD29 (Biolegend, USA, 303015), CD90 (Biolegend, USA, 328107), and CD105 (Biolegend, USA, 800505). Non-specific staining was controlled with isotype-matched antibodies. ADSCs suspensions were incubated with primary antibodies (1:50), washed, and analyzed using a flow cytometer (Agilent, USA).

**Supplementary Figure legends**

**Figure S1. Adipose derived stem cells of polydactyly (Pol-ADSCs) and macrodactyly (Mac-ADSCs)** **exhibited similar surface ADSCs markers.**

**A.** Flow cytometry analysis of cell surface marker expression in adipose derived stem cells of polydactyly (Pol-ADSCs) and macrodactyly (Mac-ADSCs).

**Figure S2. Ubiquitin mediated proteolysis ranked among the top 10 enriched KEGG pathways in Mac-AT versus Pol-AT transcriptomes.**

**A.** Bubble chart of the top 10 enriched KEGG pathways among DEGs between Pol-AT and Mac-AT (GSE298035).

**Figure S3. The full length uncropped original western blots in Figure 1 and Figure 2.**

**Figure S4. The full length uncropped original western blots in Figure 4.**

**Figure S5. The full length uncropped original western blots in Figure 5.**
