# Supplementary figures and images for: Activating PIK3CA mutation promotes overgrowth of adipose tissue via inhibiting lipophagy in macrodactyly
Source: Cell Death Dis. 2025 Oct 6;16(1):686. doi: 10.1038/s41419-025-08024-x (PMC12501352; doi:10.1038/s41419-025-08024-x)

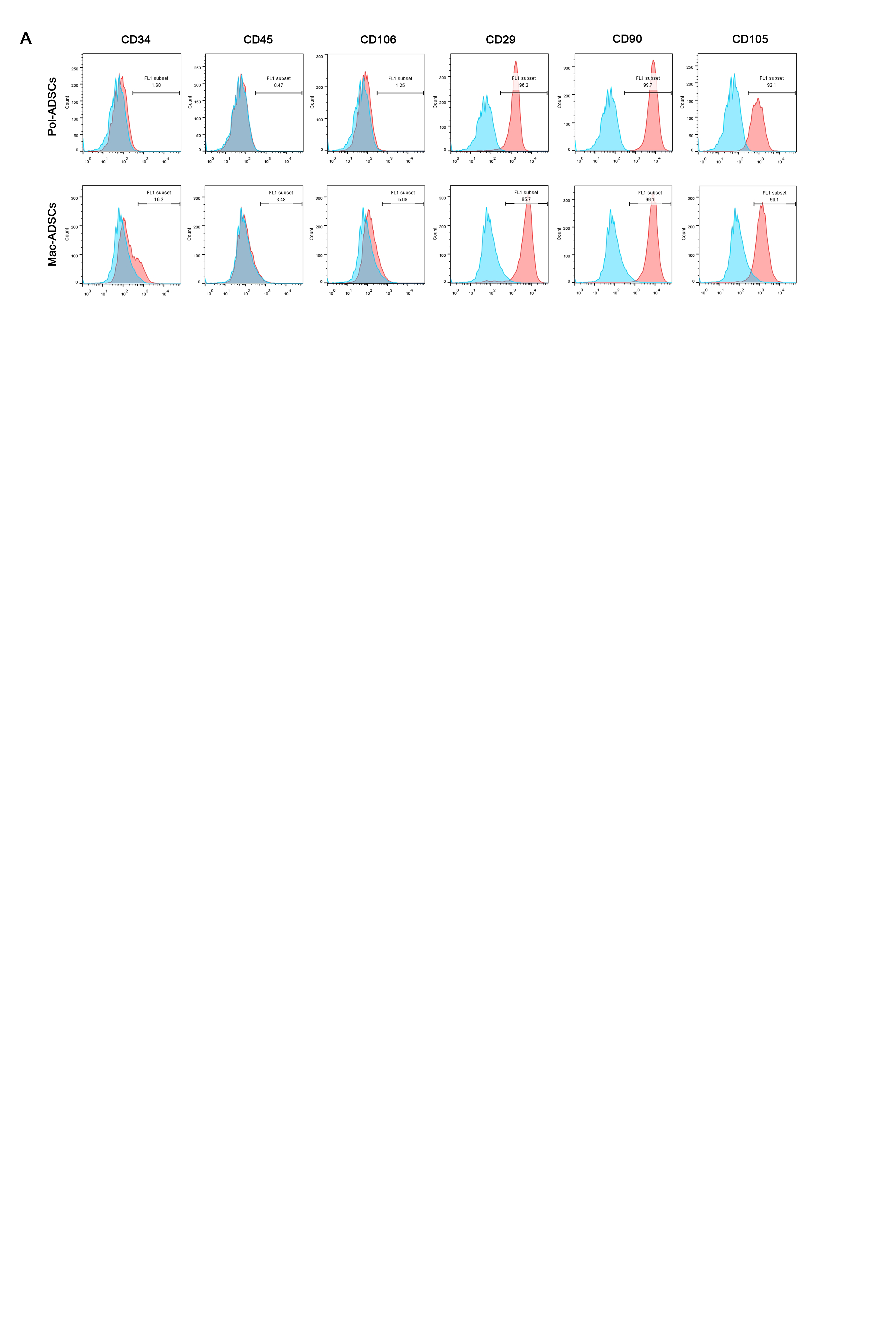

Supplement: Supplementary file 2 — Figure S1 [file 41419_2025_8024_MOESM2_ESM.tif]

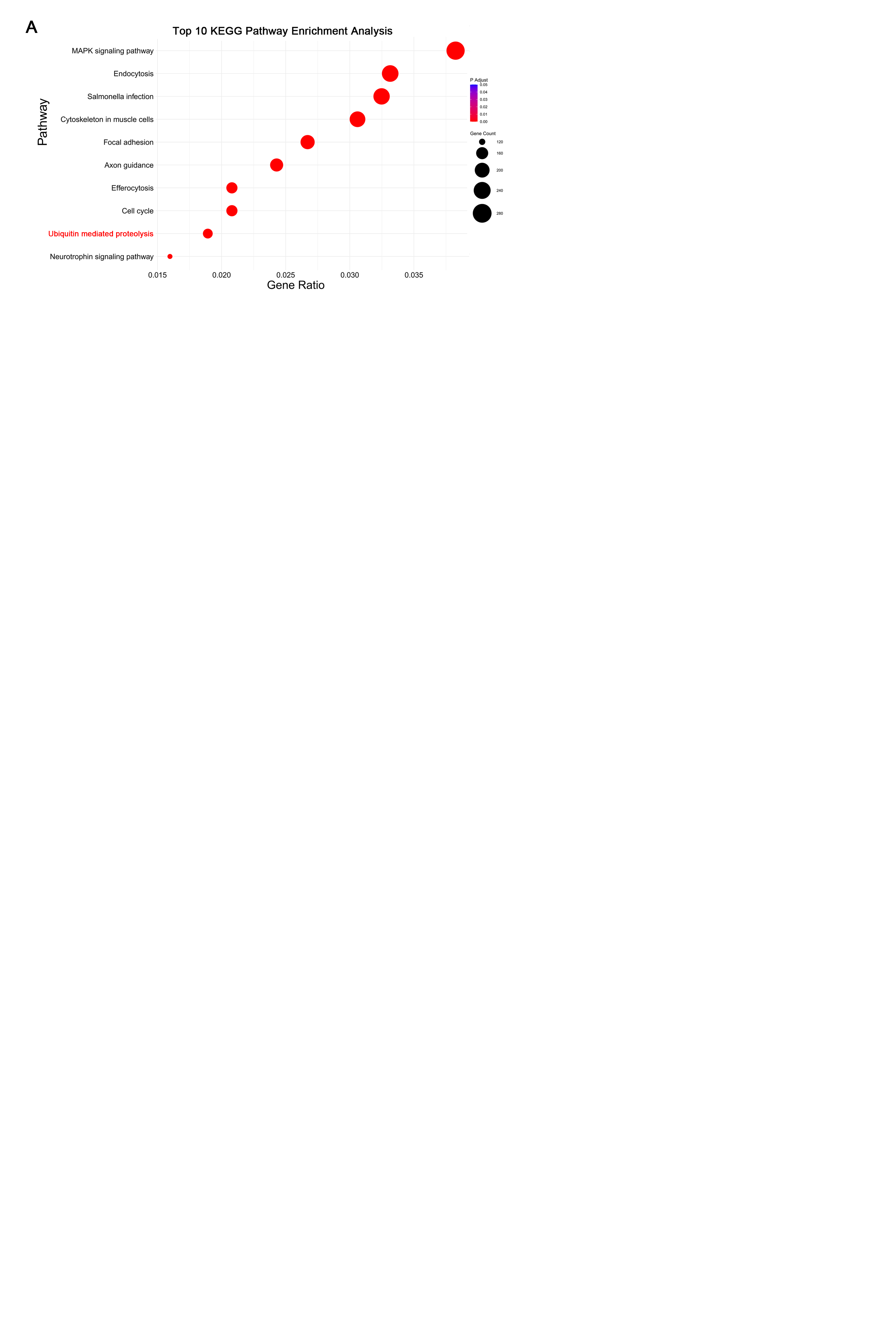

Supplement: Supplementary file 3 — Figure S2 [file 41419_2025_8024_MOESM3_ESM.tif]

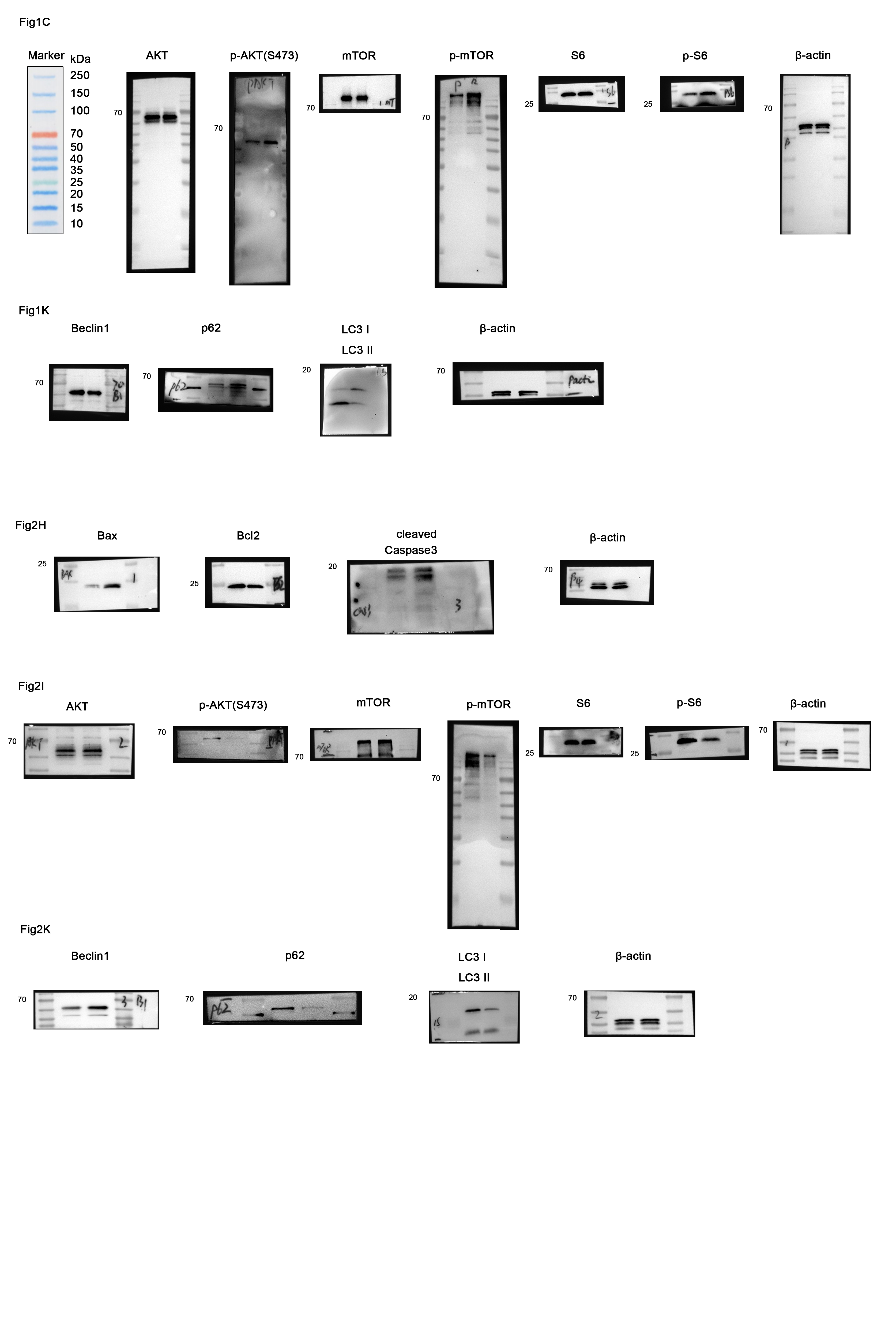

Supplement: Supplementary file 4 — Figure S3 [file 41419_2025_8024_MOESM4_ESM.tif]

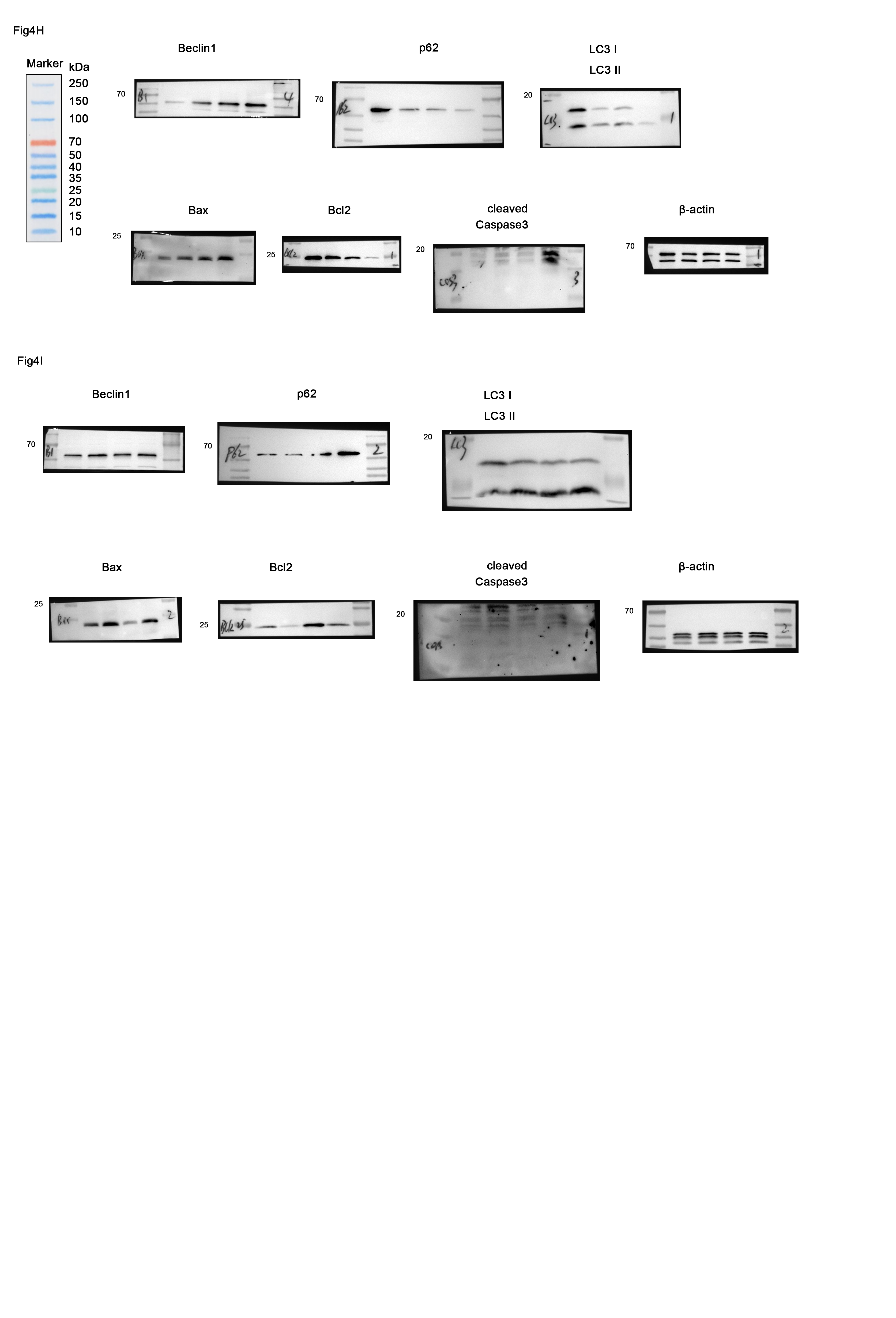

Supplement: Supplementary file 5 — Figure S4 [file 41419_2025_8024_MOESM5_ESM.tif]

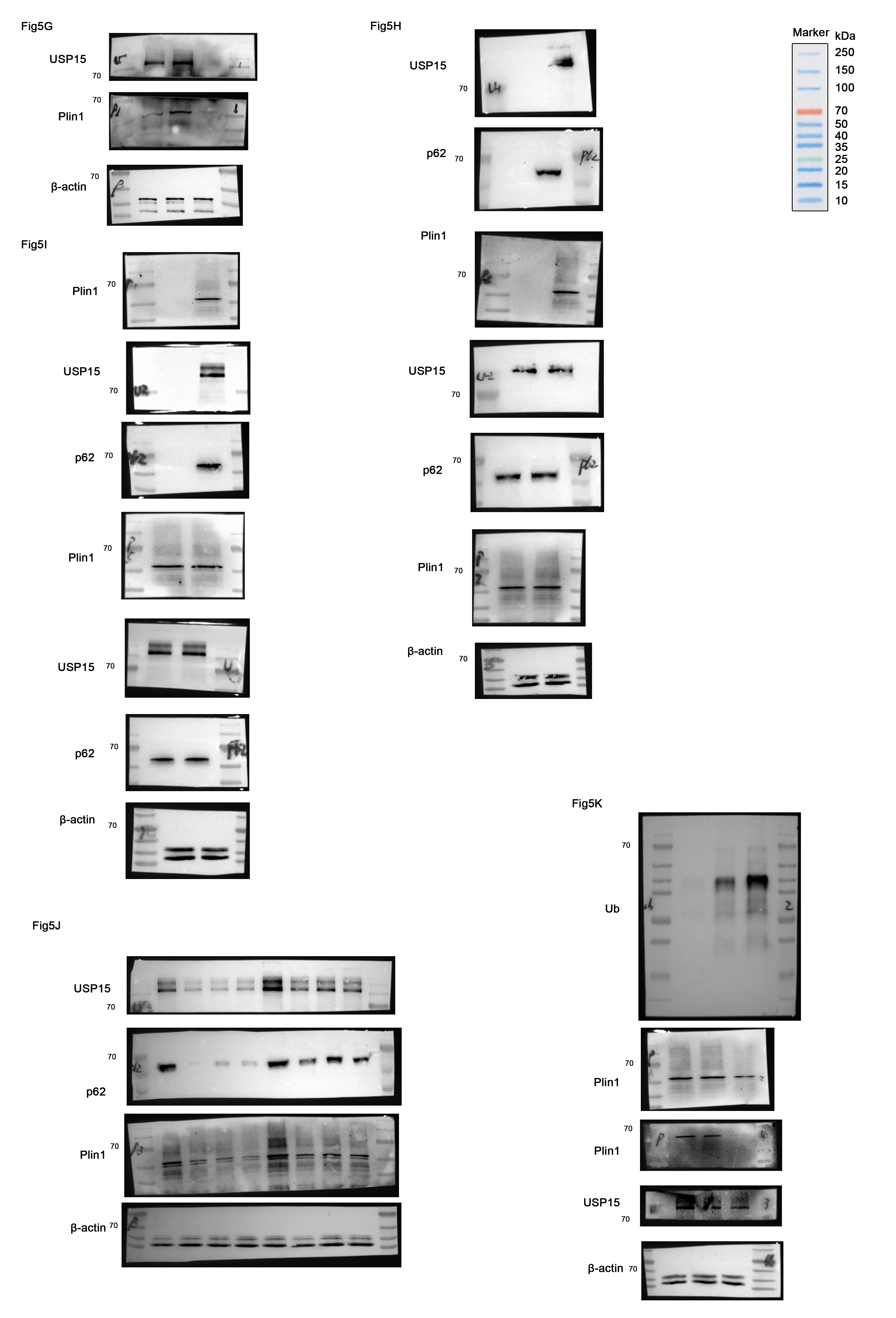

Supplement: Supplementary file 6 — Figure S5 [file 41419_2025_8024_MOESM6_ESM.tif]
