# Supplementary material for: Activating PIK3CA mutation promotes overgrowth of adipose tissue via inhibiting lipophagy in macrodactyly
Source: Cell Death Dis. 2025 Oct 6;16(1):686. doi: 10.1038/s41419-025-08024-x (PMC12501352; doi:10.1038/s41419-025-08024-x)
Supplement: Supplementary file 7 — Table S1 [file 41419_2025_8024_MOESM7_ESM.docx]

**Table S1. Quantitative PCR amplification primers**

| **Gene name** | **Forward primer** | **Reversed primer** |
| --- | --- | --- |
| GAPDH | GGAAGCTTGTCATCAATGGAAATC | TGATGACCCTTTTGGCTCCC |
| LC3B | AACGATACAAGGGTGAGAAGCA | CACTGACAATTTCATCCCGAAC |
| BECLIN1 | GGCACAATCAATAACTTCAGGC | CCGTAAGGAACAAGTCGGTATCTC |
| ATG7 | TAGTAGTGCCTTGGATGTTGGG | CAGCCCAGCAGAGTCACCATT |
| ATG5 | GGCCATCAATCGGAAACTCA | CGGGTAGCTCAGATGTTCACTC |
| E2F1 | CCGTGGACTCTTCGGAGAAC | ATCCCACCTACGGTCTCCTC |
| CDK4 | TCTGGTGACAAGTGGTGGAACA | GCAGCCCAATCAGGTCAAA |
| CDK6 | TCCCAGGCAGGCTTTTCAT | GGTCCTGGAAGTATGGGTGAGA |
| SREBP1 | TCTGGAGGCATCGCAAGC | AGCAGGTGACGGATGAGGTT |
| ACLY | CTCACTAAGCCCATCGTCTGC | ACAAACACTCCTGCTTCCTTCA |
| ACACA | GCACAATCCTTAGGGACAACATAC | ATGCCAATCTCATTTCCTCCTG |
| FASN | GAGTTCTGGGACAACCTCATCG | AGACGCCAGTGTGTGTTCCTC |
| SCD | GATGCCCCTCTACTTGGAAGAC | CAAAATAGTAGAATACCCCCCAAAGC |
| PIK3CA | AGCACCTGAATAGGCAAGTCG | TAGTTGATGAGCAGGGTTTAGAGG |
| CPT1 | GAAGTTGTTCAAGTTGGCGTCT | ATGTACGACACACCATAGCCGT |
| PLIN1 | AAGACCCTCCAGACCACCATC | ATTCGCTCTCGGGCTCCAT |
| PLIN2 | ACTGATGAGTCCCACTGTGCTG | AGGCAGCATTGCGGAACAC |
| PLIN3 | AGCGGCAGGAACAGAGCTACTT | GATCAACGCCTTGCTTGACAGT |
| PLIN4 | CCAGCGGGCATTTGAACA | CTTTTCAATCAGCCTGAAGCAGT |
| PLIN5 | CTTCCCTTTCTCCAGCAACC | TCCTCCACCGAACCCACTT |
| USP1 | GGAGTTTGATTGTTATGGTGGTGG | CTGTCGTTAGTTGGCTTTGTGCT |
| USP12 | CTCTTCCATAGCATAGCCACTCA | TGGGCATCTTGTTGCATGTAGT |
| USP13 | ACCTCCATGGGATTTCAGCG | ACTCTAGGTCCTTCGGGCTT |
| USP15 | TCCTGCTCTTGAGAATGTGCC | TGTCCCGTGAGTTGACCTTTG |
| USP16 | GCTTCGCTACTTATTGGATGGG | GGGAGGGACAAATCAAGGAAA |
| USP24 | ACGCGGAGAAGAATGATGAGAA | TCATCAGACTCGGAAAGACCAA |
| USP31 | AGCCTCCTCTGAAGCCACCAT | GCGACAGTCCCAGACAGAGGT |
| USP34 | AGAGTGCAGCTCGTAACATGGC | ACTCAATCCAGCCAACCTCATAG |
| USP37 | GCGAAACAAAGCCGCCTAA | GGCTGCAGGAAGTCTGTTTTG |
| UCHL5 | CAGGATGTCCATTTAGGCGAG | TTGCTCAGTGCCAAGCCTTT |
