# Supplementary material for: Activating PIK3CA mutation promotes overgrowth of adipose tissue via inhibiting lipophagy in macrodactyly
Source: Cell Death Dis. 2025 Oct 6;16(1):686. doi: 10.1038/s41419-025-08024-x (PMC12501352; doi:10.1038/s41419-025-08024-x)
Supplement: Supplementary file 8 — Table S2 [file 41419_2025_8024_MOESM8_ESM.docx]

**Table S2. siRNA sequences used for USP15 Knockdown**

| **siRNA name** | **Sense strand** | **Antisense strand** |
| --- | --- | --- |
| siNC | UUCUCCGAACGUGUCACGU TT | ACGUGACACGUUCGGAGAA TT |
| siUSP15-1 | CUGCAAAGUAGAAGUAUAUdTdT | AUAUACUUCUACUUUGCAGdTdT |
| siUSP15-2 | CCUGGACCCAUUGAUAACUdTdT | AGUUAUCAAUGGGUCCAGGdTdT |
| siUSP15-3 | CCCUGGUAUUGUCCGAAUUdTdT | AAUUCGGACAAUACCAGGGdTdT |
